# A versatile platform for graphene nanoribbon synthesis, electronic decoupling, and spin polarized measurements†

**DOI:** 10.1039/d2na00668e

**Published:** 2023-02-02

**Authors:** Aleš Cahlík, Danyang Liu, Berk Zengin, Mert Taskin, Johannes Schwenk, Fabian Donat Natterer

**Affiliations:** a Department of Physics, University of Zurich Winterthurerstrasse 190 CH-8057 Zurich Switzerland ales.cahlik@physik.uzh.ch fabian.natterer@physik.uzh.ch; b Institute of Physics, EPFL Station 3 CH-1015 Lausanne Switzerland

## Abstract

The on-surface synthesis of nano-graphenes has led the charge in prototyping structures with perspectives beyond silicon-based technology. Following reports of open-shell systems in graphene-nanoribbons (GNRs), a flurry of research activity was directed at investigating their magnetic properties with a keen eye for spintronic applications. Although the synthesis of nano-graphenes is usually carried out on Au(111), the substrate is difficult to use for electronic decoupling and spin-polarized measurements. Using a binary alloy Cu_3_Au(111), we show possibilities for gold-like on-surface synthesis compatible with spin polarization and electronic decoupling known from copper. We prepare copper oxide layers, demonstrate the synthesis of GNRs, and grow thermally stable magnetic Co islands. We functionalize the tip of a scanning tunneling microscope with carbon-monoxide, nickelocene, or attach Co clusters for high-resolution imaging, magnetic sensing, or spin-polarized measurements. This versatile platform will be a valuable tool in the advanced study of magnetic nano-graphenes.

## Abstract 2

Decoupling oxide layers, magnetic cobalt islands, and on-surface synthesis of nano-graphenes, paired with easy spin-polarization.

## Graphical abstract

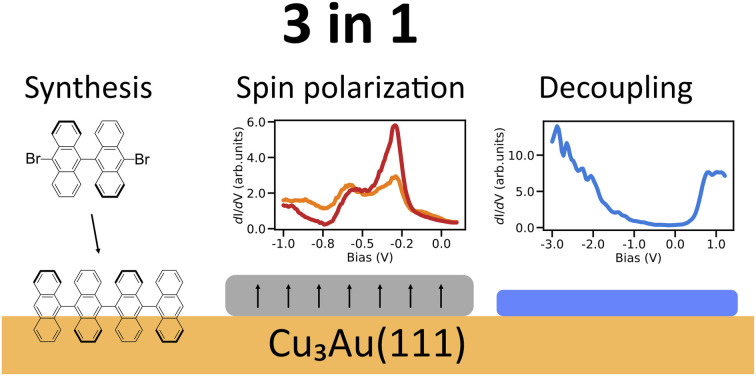

## Introduction

Through experience, we have come to accept that one cannot always have one's cake and eat it, too. Unfortunately, this is also true in scientific endeavors where it may even be self-induced. This partially originates in the way investigations grow from the bottom-up, expanding on previous progress and following a curiosity driven path. This style of research works well until a top-down challenge arises that may be incompatible within the so far used framework. We see parallels to this conundrum in research following the pioneering studies on on-surface synthesis of graphene nanoribbons (GNRs)^1^ and chemically tailored nano-graphenes.^2^ For their synthesis, an overwhelming number of contributions have converged on the coinage metals gold^3–15^ or copper.^2,16–19^ Although both provide simple preparation, gold is deemed favorable for tailored synthesis because it reliably facilitates polymerization directly at the halogenated carbon site. For example, the on-surface reaction of 10,10′-dibromo-9,9′-bianthracene (DBBA) results in the growth of straight *N* = 7 armchair graphene nanoribbons (7-AGNRs) on Au(111),^1^ whilst on Cu(111) partially chiral nanoribbons are formed.^17,20^ However, with the emergence of magnetic signatures in carbon-based systems,^5–7,11,12,15,21^ further investigation may depend on our ability to provide orbital-imaging, spin-polarization and decoupling from itinerant electrons that are hard to simultaneously satisfy for either substrate. When looking for model-systems of spin-polarization, we notice in cobalt nanoislands on copper^22,23^ a similar monoculture with a heavy focus on this platform despite it being plagued by the rapid intermixing of Co and Cu at room temperature,^24^ which precludes its use for thermally induced nano-graphene formation. Perhaps more liberty and flexibility are found in investigations of decoupling layers such as Cu_2_N/Cu,^25,26^ MgO/Ag,^27,28^ or NaCl^3,29^ because they appear motivated by top-down questions about the pristine properties of single atoms or molecules. A natural question is therefore to ask whether tailored on-surface synthesis, spin-polarization, and decoupling are exclusive and if not whether we can identify systems that simultaneously host the properties that made one specific platform so popular.

Here we establish with Cu_3_Au such a system that combines the key properties of the coinage metals Au and Cu. We show the preparation of clean Cu_3_Au(111) surfaces and the growth of copper oxide decoupling layers. Using the prototypical halocarbon precursor DBBA, we demonstrate its polymerization and cyclodehydrogenation into 7-AGNR. We show that the latter step can be temperature induced and possibly also be achieved *via* deliberate tip-manipulation using a scanning tunneling microscope (STM). We use a high-resolution STM with carbon-monoxide (CO) functionalized tips to verify the successful synthesis into 7-AGNRs and observe a spontaneous formation of GNRs exhibiting zero-bias peaks, suggestive of a Kondo resonance. In order to demonstrate spin-polarization on Cu_3_Au, we prepare cobalt nanoislands that are thermally and magnetically stable and serve as an easy means to produce spin-polarized tips. Together, these salient features demonstrated in our work equip the community with a versatile platform to tackle pressing top-down questions in surface science and in the advanced investigation of carbon-based systems.

## Results and discussion

### Cu_3_Au substrate and its oxidation

**Fig. 1:**
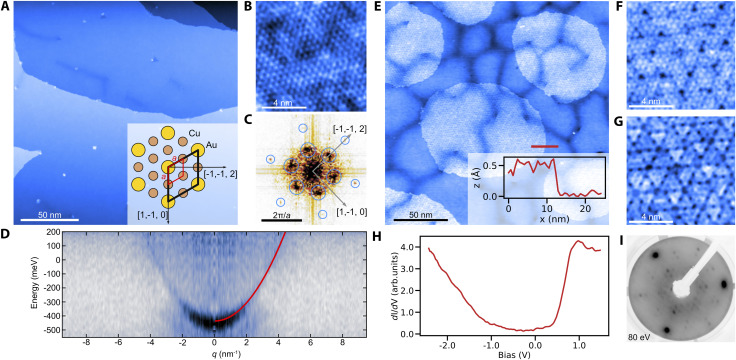
Properties of Cu_3_Au(111) and copper oxide overlayers. (A) Overview topography of clean Cu_3_Au(111) terraces (*V* = 1 V, *I* = 140 pA) with the ideal L1_2_ surface termination indicated in the inset, partially visible by the atomically resolved image in (B) and emphasized in its Fourier transform in (C), showing the atomic lattice (small circles) and the (2 × 2) supercell (large circles). (D) The surface hosts a nearly-free electron like surface state with an effective mass of m*/*m*_e_ = 0.31 ± 0.02 as determined by the parabolic fit (red-line) to the dispersion. The dispersion plot was produced from the measurement of the energy dependent local density of states on a field-of-view of 105 nm (drive frequency 1600 Hz, modulation amplitude 0.5 V, offset −0.25 V). (E) Topographic image of the Cu_3_Au surface with oxide patches after exposing it to molecular oxygen. Inset shows the apparent height of the oxide patch relative to the Cu_3_Au surface (marked by the red line) (−0.5 V, 50 pA). (F) and (G) Threefold symmetric contrast in zoomed images of the oxide patch for two different bias values (*V* = −0.3 V and *V* = 0.5 V respectively). (H) Point tunneling spectroscopy of the oxide phase showing a gap-size of about 1.3 V. (I) LEED image of the oxidized substrate at 80 V beam energy showing spots in addition to the Cu_3_Au lattice.

We prepare clean surfaces of Cu_3_Au following previous work,^30^ using standard surface cleaning procedures described in the Experimental section. Fig. 1A shows an STM overview topography of a Cu_3_Au(111) surface termination with large terraces routinely exceeding 150 nm width and separated by monoatomic steps. High-resolution imaging using a carbon-monoxide functionalized tip shows the atomic lattice and the (2 × 2) supercell of the L1_2_ ordered phase (Fig. 1B and C).^31^ Similar to the pure coinage metals, Cu_3_Au(111) exhibits a nearly-free electron like surface state with an effective mass m*/*m*_e_ = 0.31 ± 0.02 and a band onset of *E*_0_ = 0.42 eV below the Fermi level when measured using quasiparticle interference imaging,^32^ comparable to the observed value in previous work using angle-resolved photoelectron emission spectroscopy.^33^ The darker line-features in Fig. 1A and E occasionally form complete hexagonal networks with a long-range periodicity of about (36 ± 3) nm (Fig. S1†). We propose that these networks are associated with the previously reported 29 nm surface reconstruction, which had been noticed as faint spots in low-energy electron diffraction (LEED) experiments and described as a “herringbone-like” reconstruction^30^ (for details see the ESI†).

Having established the preparation of clean surfaces of Cu_3_Au and verified their properties, we proceed to demonstrate the growth of copper oxide overlayers. We deliberately dose small amounts of molecular oxygen while annealing the crystal (see the Experimental section) to form sub-monolayer patches of an oxidized substrate (Fig. 1E). The threefold symmetric contrast (Fig. 1F and G) is reminiscent of cuprous oxide in previous reports for Cu_2_O/Pt(111).^34^ Furthermore, the spectroscopic signatures (Fig. 1H) of the oxide patch show a characteristic bandgap of about 1.3 eV in good agreement with observations for Cu_2_O/Au(111).^35^ The oxide formation is further confirmed by a distinct change in the LEED pattern after oxygen exposure (Fig. 1I). We also observe the formation of oxide layers from interstitial oxygen that naturally segregates towards the surface during repeated and prolonged annealing at temperatures above 600 °C. In this case, two coexisting phases are formed (Fig. S2†). Besides the threefold symmetric phase observed after the deliberate oxygenation, a new stripe-like phase emerges exhibiting a similar bandgap in tunneling spectroscopy. We have found no direct precedent for this phase, but Cu_2_O is known to constitute a wealth of structures including phases of reduced symmetry, such as in the Pt(111) system.^36^

### Synthesis of nano-graphenes

**Fig. 2:**
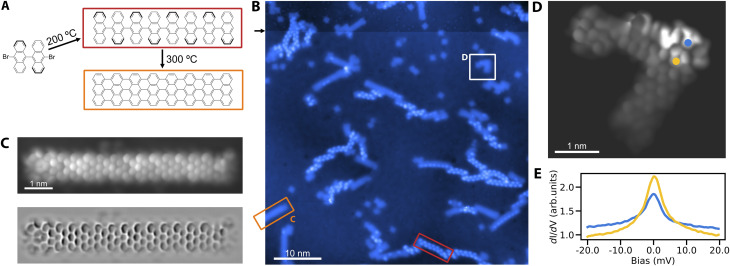
On-surface synthesis of nano-graphenes on Cu_3_Au(111). (A) Two step 7-AGNR annealing scheme from DBBA precursor molecules. (B) Overview topography after dosing DBBA onto the substrate kept at 200 °C and post annealing at 300 °C (*V* = 50 mV, *I* = 30 pA). While DBBA readily polymerizes according to the scheme in (A), the cyclodehydrogenation was interrupted after 15 minutes annealing at the higher temperature to leave some chains polymerized but still hydrogenated (marked by the red rectangle). The black arrow marks a spontaneous tip functionalization with a CO molecule. (C) Upper: High-resolution STM image of a fully formed *N* = 7 armchair GNR using a carbon-monoxide functionalized tip. Lower: Laplace-filtered version highlighting the GNR structure. (D) High-resolution STM image of a defective GNR measured using a carbon-monoxide functionalized tip. (E) A zero-bias peak in tunneling spectroscopy, suggestive of a Kondo resonance, obtained above a spontaneously formed defective GNR [positions marked by dots in panel (D)].

We proceed to verify the utility of Cu_3_Au(111) for the on-surface synthesis of nano-graphenes. The most prominent example is the growth of GNRs from DBBA precursor molecules on different coinage metal substrates.^1,17,20^ We establish the growth of straight 7-AGNRs on Cu_3_Au(111) by pursuing the common two-step formation procedure (Fig. 2A). To that end, we deposit the DBBA precursor molecules on the substrate kept at 200 °C, leading to the dehalogenation and subsequent polymerization of the bi-anthracene moieties into one-dimensional chains. The second annealing step at a higher temperature of 300 °C yields through cyclodehydrogenation the fully formed GNRs (Fig. 2B). To obtain a peek into the two-step synthesis, we interrupt the high temperature annealing after 15 minutes to ensure the coexistence of fully formed GNRs and the polymerized but still hydrogenated chains. The polyanthrylene chains can be distinguished from the GNRs by their bead-like protrusions and larger apparent-height as reported in previous work.^1^ We verify the structure of the 7-AGNRs using high-resolution STM imaging with a CO functionalized tip (Fig. 2C). The image clearly reveals the straight 7-AGNR structure corresponding to the nanoribbon formation on Au(111)^1^ in contrast to the growth of partially chiral nanoribbons from DBBA on Cu(111).^17^ We tentatively attribute this behavior to: (1) a vertical relaxation of Au atoms of the surface layer above the Cu atoms,^37^ exposing them more for Ullmann coupling reactions, and (2) a significant enrichment of the surface layer by Au atoms compared to the ideal Cu_3_Au(111) surface.^30^

In addition to pristine 7-AGNRs, we also observe the spontaneous growth of defective ribbons (Fig. 2D). The interest in GNR defects lies in their connection to single electron spins in open-shell structures exhibiting magnetic signatures.^15^ Similarly, we observe the characteristic zero bias peaks in scanning tunneling spectroscopy for several defective structures, suggestive of Kondo resonances (Fig. 2E) – an established feature of the stabilized radical.

Remarkably, when scanning at elevated bias, we notice instabilities in the protrusions of polyanthrylene chains, indicating a mechanism for their deliberate dehydrogenation, similar to previous observations.^38^ We accordingly use the tip of the STM to selectively strip-off hydrogen from selected parts of the polymerized chains (Fig. S3†). Although the thus formed ribbons show a similar apparent height than the thermally produced GNRs, they exhibit more irregularities and variation in their topographic and electronic structures. Whether tip mediated GNR formation is a matter of tuning the manipulation parameters or intrinsically limited, could become the subject of further study.

### Magnetic properties of cobalt nanostructures and spin-polarization

**Fig. 3:**
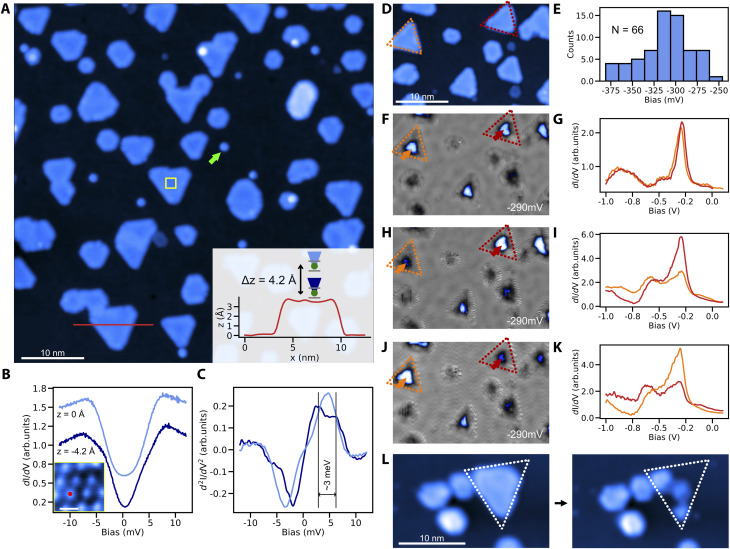
Growth of cobalt islands on Cu_3_Au and facile preparation of spin-polarized tips. (A) Overview topography of two-monolayer high Co islands that were grown on Cu_3_Au at 200 °C with the line profile across the island shown in the inset (*V* = 50 mV, *I* = 5 pA). The circular and triangular islands have different stacking with respect to the substrate. (B) Tunneling conductance spectrum and (C) its derivative taken with a nickelocene functionalized tip for two different heights [Δ*z* = 4.2 Å, inset of (A)] above a Co atom in the middle of an island [marked in the inset of (B)]. The spectrum taken closer to the Co atom shows a splitting of ∼3 meV, corresponding to the exchange field in the order of 13 T (39). (D) Topography image containing two similarly sized islands (dotted lines) with identical stacking used for the magnetic characterization of their magnetic bistability and spin-polarization. (E) Histogram of the d-level peak position with a mean of −310 mV as determined from 66 randomly picked triangular islands. (F) Closed loop conductance image taken at −290 mV, measured with a spin-averaging tip and the corresponding point spectra (G) of the orange and red islands showing no contrast difference (10 mV modulation). (H and I) Conductance map and tunneling spectra measured over the same region with a spin-polarized tip (10 mV modulation). The intensity of the d-level shows a clear difference that is attributed to two distinct magnetic orientations between the two islands with respect to the tip magnetization and that is also visible as contrast variation in the conductance map. (J and K) Conductance map and tunneling spectra with a spin-polarized tip whose magnetization spontaneously switched, leading to a reversed contrast between the two magnetically differently oriented islands. (L) Spin-polarized tips can be produced by transferring an island from the substrate to the tip as shown by the before and after topographic images (*V* = 400 mV, *I* = 50 pA).

We finally demonstrate the sub-monolayer (ML) growth of cobalt nanoislands on Cu_3_Au(111) and characterize their magnetic signatures. In contrast to the fast intermixing that is typical of Co/Cu(111) already at room-temperature, the Co/Cu_3_Au interface is thermally robust. We do not observe signs of intermixing for substrate temperatures up to about 300 °C. After deposition of 0.3 ML of Co at about 200 °C, we observe the growth of round and triangular Co islands of ∼3.4 Å apparent height (Fig. 3A). Compared to the cobalt islands on Cu(111), the islands appear smaller with the edge length spanning from 15 nm to a few nm, attributed to the larger lattice mismatch (5% for Co–Cu_3_Au and 2% for Co–Cu).^39^ At the same time, the islands are larger than cobalt clusters on Au(111) (13% lattice mismatch for Co–Au), where edge effects lead to noncollinear magnetization.^39^

For the magnetic characterization, we focus on triangular islands due to their resemblance with the model system Co/Cu(111).^22^ To that end, we use nickelocene (NiCp_2_) molecules, whose spin-excitation from the *S* = 0 ground state to the *S* = ±1 excited state is sensitive to the magnetic or exchange field applied along the molecular axis.^40,41^ At zero-field, both ground state to *S* = ±1 transitions are degenerate but in an applied field, the Zeeman splitting separates the single step in the tunneling conductance into two. Exposing a NiCp_2_ functionalized tip to the exchange field of a Co island on Cu_3_Au results in a clearly observable splitting of the step feature in STS when we move the NiCp_2_ tip closer to the Co island (Fig. 3B and C). The proximity leads to an enhanced exchange field and concomitantly to a larger Zeeman splitting. This exchange interaction related shift in the spin-excitation step is equivalent to previous reports for NiCp_2_ measured against Co islands on Cu(111).^40^

To establish the magnetic bistability of our Co islands, we select two triangular islands of identical stacking and similar size to avoid structural ambiguities (Fig. 3D). To further ensure their electronic equivalency without strain induced effects,^39,42^ we measure the tunneling spectra with a spin-averaging tip. The two selected islands are spectroscopically equivalent and share the same d-level position at −290 mV of comparable intensity (Fig. 3F and G). We note that the average d-level position at −310 mV in tunneling spectroscopy (see the histogram for tunneling spectra taken above 66 random triangular islands in Fig. 3E) is comparable with the reported peak position for Co on Cu(111),^39^ although the shift towards lower energies could be expected due to larger lattice mismatch between cobalt and Cu_3_Au. We tentatively attribute this to edge induced effects on the peak position due to the smaller island size.^39^ Using a spin-polarized tip on the same two islands, the intensity of the d-level peak becomes distinct between the orange and red islands (Fig. 3H and I), showing an unequivocal signature of tunnel magnetoresistance stemming from the different magnetic orientations of the two islands with respect to the magnetization of the tip. This is further supported by a contrast reversal between the two islands after a spontaneous switch of the tip magnetization (Fig. 3J and K).

For completeness, we finally demonstrate how to readily produce spin-polarized tips by simply picking up a Co island from the Cu_3_Au substrate which transfers Co to the tip apex (see the Experimental section for details). This procedure leaves the neighboring structures intact (Fig. 3L), causing minimal disruption to local details.

## Discussion and outlook

When we look at the overall properties of Cu_3_Au, we identify interesting opportunities and perspectives. The most notable behavior is the thermal stability of the interface to increased temperature, which may be crucial for temperature driven on-surface synthesis. This holds, even in the presence of the copper oxide overlayer and the magnetic Co islands; the latter being thermally stable up to 300 °C. The value of high temperature stability is immediately clear when we compare it to the requirements for dehalogenation and cyclodehydrogenation as elementary steps in Ullmann coupled systems (Fig. 2A). Our work thus combines the controlled on-surface synthesis of GNRs known from Au(111) with the magnetism typical of Co/Cu(111). Preliminary experiments prove that Co islands and GNRs can coexist (Fig. S4†). The absence of the short-range herringbone reconstruction on Cu_3_Au(111) is helpful in reciprocal space analyses of synthesis steps and the possibility for magnetic Co islands offers facile spin-polarization and magnetic references for the study of open-shell GNRs. In addition, the copper oxide may serve as an alternative decoupling layer that isolates molecular structures synthesized *via* Ullmann coupling reactions from itinerant electrons. Additional surface chemistry can become active at elevated temperatures (see the discussion in the ESI and Fig. S5†) and may need to be scrutinized against the requirements of one's experiment. The Cu_3_Au system can also stabilize a lattice matched copper-nitride for which electronic decoupling was successfully demonstrated with single atoms.^43,44^

The tip functionalization on Cu_3_Au is straightforward which is valuable for the verification of synthesis steps or to enhance certain contrast modes. We demonstrate this with the routine use of CO, NiCp_2_, and Co cluster functionalized tips for high-resolution imaging, magnetic imaging, and spin-polarization, respectively. The facile tip-functionalization promotes further work on magnetism relying on direct tunneling^45^ or STM enabled electron-spin resonance experiments.^28^ In addition to functionalization, we also show how the tip can be used to selectively dehydrogenate polymerized molecules. This invites further work utilizing defects or barriers in circular or one-dimensional polymerized carbon structures.

## Experimental

### Sample preparation

The Cu_3_Au(111) crystal was purchased from Surface Preparation Laboratory. We mount it on a ferromagnetic sample plate to allow for the attachment of permanent magnets for field-dependent studies^46^ and prepare atomically clean surfaces analogous to the literature^30^ by repeated cycles of Ar^+^ ion bombardment and annealing (3 keV, 0.8 μA cm^−2^, 550 °C). To verify the impact of the order/disorder transition, we perform extended annealing procedures (16 hours at 340 °C) to promote ordering of the L1_2_ structure.^31^ We find no evidence that the ordering level influences the GNR formation or the cobalt island growth. The oxidized surface was prepared by annealing a clean Cu_3_Au substrate at 400 °C while exposing to an 8 × 10^−7^ mbar O_2_ atmosphere for 20 minutes. We grow cobalt islands from thoroughly degassed Co rods using a commercial e-beam evaporator (Focus EFM3) at sample temperatures from 60 °C to 300 °C. Increasing the substrate temperature beyond 300 °C results in visible Co intermixing at the step edges, emergence of vacancies and sinking of the Co islands into the substrate. For the GNR growth, we dose commercially available 10,10′-dibromo-9,9′-bianthracene precursors (Chemie Brunschwig AG, CAS: 121848-75-7) using an effusion cell (Kentax, TCEorg-3BSC) or a home-made evaporator at a source temperature of 160 °C.

### Tip functionalization

For high-resolution imaging, we functionalize the tip with a carbon monoxide molecule that we pick off the substrate spontaneously during scanning (Fig. 2B) or by stabilizing the tip above the molecule (100 mV, 10 pA), switching off the feedback loop and increasing the bias to 3 V. We dose nickelocene (NiCp_2_) from a commercially available powder (Chemie Brunschwig AG, CAS: 1271-28-9) directly onto the cooled sample in the microscope head. The nickelocene tip functionalization is performed with feedback loop off, by manually approaching the tip to the molecule at 20 mV bias until a sharp jump in the current channel is observed. For spin-polarized measurements, we deliberately transfer a Co island from the Cu_3_Au substrate to the tip. This is done by approaching the island from 20 pA, 50 mV setpoint by 400–500 pm while applying 2–2.5 V bias.

### STM measurements and tunneling spectroscopy

All experiments are performed in ultra-high vacuum using a commercial STM (CreaTec Fischer & Co. GmbH) operating at about 4 K. Our tip is made from a mechanically cut PtIr wire and sharpened by gently plunging it into the Cu_3_Au sample until we notice sharp step edges in topographic scans. For point-spectroscopy, we use a conventional lock-in technique at a frequency of 932 Hz. For the surface state mapping, we use a multifrequency lock-in amplifier (Intermodulation Products SA, MLA-3) as described in ref. 32.

## Conclusions

In summary, we demonstrate the unique utility of the binary alloy Cu_3_Au for nanographene synthesis, electronic decoupling and magnetic characterization. Our work enables advanced investigation of open-shell carbon systems using known on-surface chemistry steps required for Ullmann coupling while preserving magnetic structures and decoupling layers. We also show the substrate's versatility for diverse SPM tip-functionalization used for magnetic sensing, high-resolution imaging, and spin-polarized tunneling, and demonstrate a deliberate and bond-specific dehydrogenation of precursor molecules. Together, our work offers a dependable foundation for advanced characterization of nanographenes.

## Data availability

All data are available in the main text, or the ESI† can be found in the Zenodo repository, https://doi.org/10.5281/zenodo.7034378.

## Author contributions

Conceptualization: AC and FDN, methodology: AC, FDN, and JS, formal analysis: AC, DL, and FDN, investigation: AC, BZ, DL, FDN, and MT, visualization: AC and DL, data curation: AC, writing—original draft: FDN, writing—review & editing: AC and FDN, supervision: FDN and AC, project administration: FDN, and funding acquisition: FDN, AC, and DL.

## Conflicts of interest

There are no conflicts to declare.

## Supplementary Material

NA-005-D2NA00668E-s001
