# Supplementary material for: A versatile platform for graphene nanoribbon synthesis, electronic decoupling, and spin polarized measurements
Source: Nanoscale Adv. 2023 Feb 2;5(6):1722–8. doi: 10.1039/d2na00668e (PMC10012868; doi:10.1039/d2na00668e)
Supplement: NA-005-D2NA00668E-s001 [file NA-005-D2NA00668E-s001.pdf]

Electronic Supplementary Material (ESI) for Nanoscale Advances.
This journal is © The Royal Society of Chemistry 2023

# Electronic Supplementary Information

## A versatile platform for graphene nanoribbon synthesis, electronic decoupling, and spin polarized measurements

Aleš Cahlík*, Danyang Liu, Berk Zengin, Mert Taskin, Johannes Schwenk, Fabian Donat Natterer*

*Corresponding authors
Email: ales.cahlik@physik.uzh.ch, fabian.natterer@physik.uzh.ch

### Supplementary Text

Superstructure on $Cu_3Au$

We observe the occasional formation of complete hexagonal networks on the surface of $Cu_3Au(111)$ with a long-range periodicity. The lowest point of the dark-line regions is approximately 60 pm below the $Cu_3Au$ surface (bright regions) when measured with nc-AFM. To highlight the extent of those networks over large distances, we show in Figure S1A a topographic image with a connectivity in excess of 400 nm. For clarity, we present a levelled image with a zoomed Fourier transform inset. The periodicity of these networks (36 ± 3) nm is of similar order than the previously reported 29 nm surface reconstruction observed in electron diffraction experiments[30], which the authors had labelled as herringbone reconstruction. Despite our use of widely different sample preparation and cleaning routines, we have not found a distinct cause promoting the formation of the hexagonal network. Notably, we have not observed any influence of its presence on the oxygen or cobalt island growth.

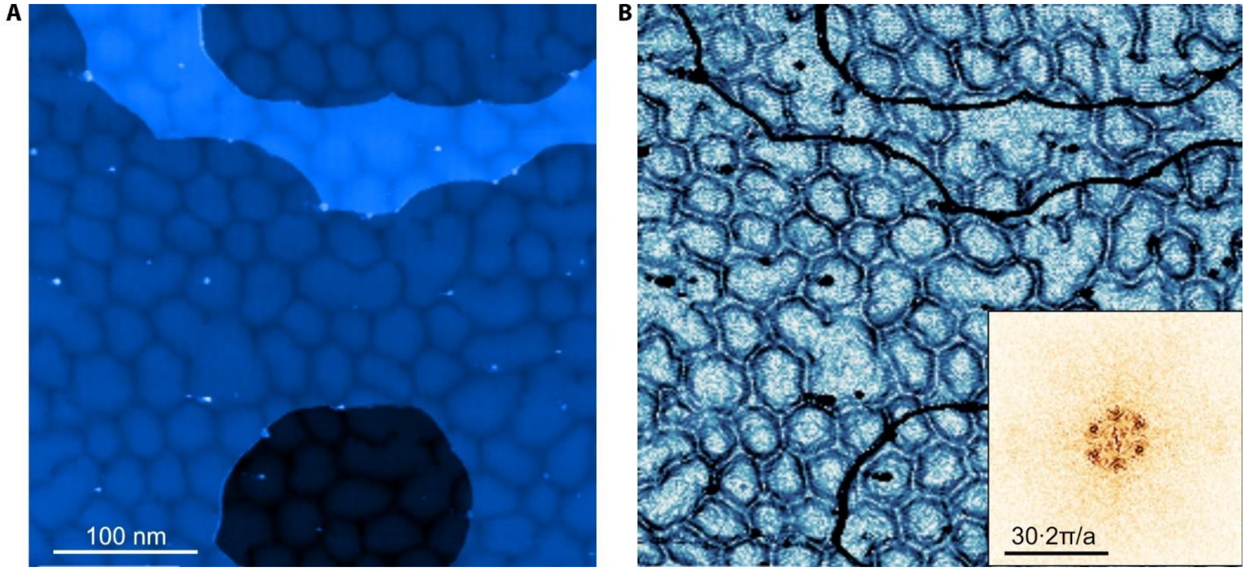


**Fig. S1 (A)** Overview topographic image showing the long-range hexagonal networks formed on $Cu_3Au(111)$ surface and its **(B)** flattened version. The inset shows a zoom of its respective Fourier transform to highlight the cell periodicity of (36 ± 3) nm**.**

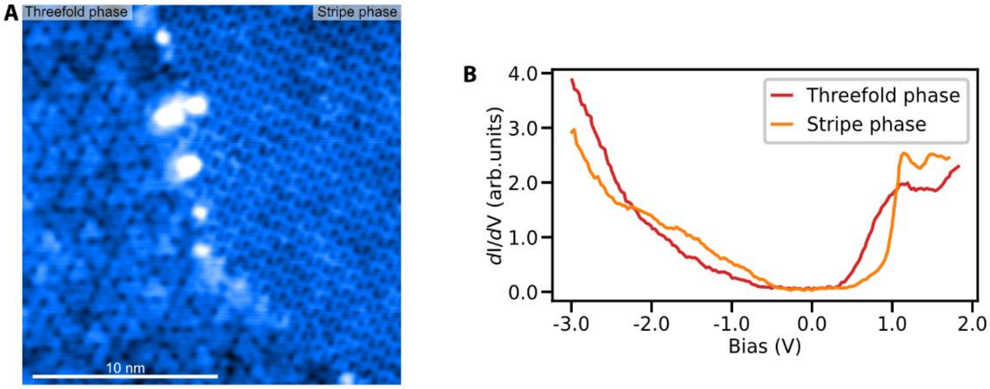


**Fig. S2 (A)** Topographic image of coexisting copper oxide phases with stripe-like and threefold symmetry that were created from interstitial oxygen that naturally segregates towards the surface during repeated annealing. (*V* = 500 mV, *I* = 100 pA). **(b)** Point STS of the two phases showing a gap-size of approximately 1.3 V.

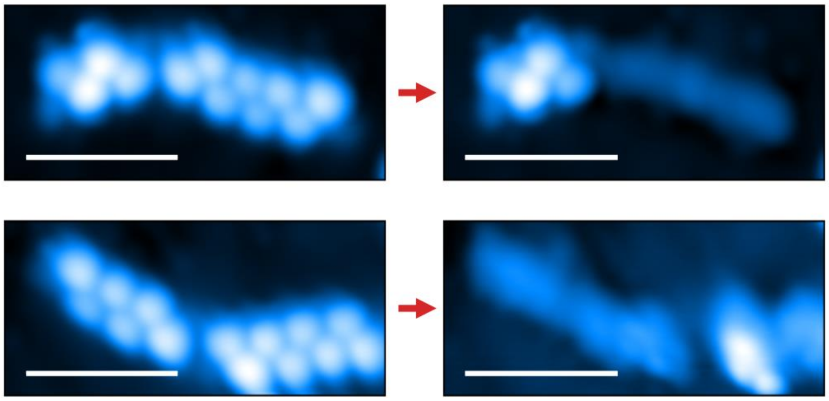

**Fig. S3** The tip of the STM can be used for targeted and deliberate dehydrogenation as shown by the topographies before and after applying current pulses on a polymerized chain (*V* = 3.5 V, *I* = 20 nA). Scalebar 4 nm.

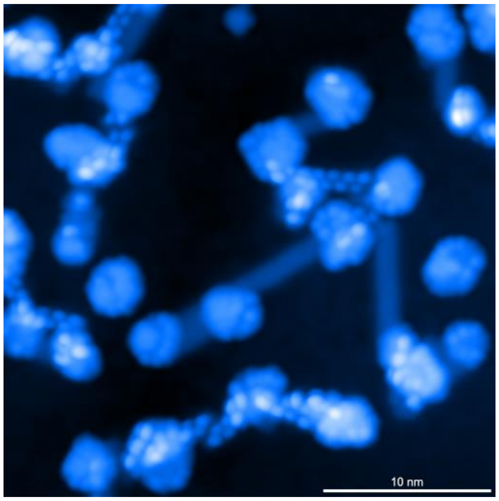


**Fig. S4** Overview topography STM topography of cobalt islands deposited after thermally induced polymerization and partial cyclodehydrogenation of the DBBA precursor on $Cu_3Au(111)$.

7-AGNR and Oxide

In our preliminary experiments towards the combination of GNR growth and surface oxidation, we first oxidized the surface and subsequently attempted DBBA polymerization and cyclodehydrogenation. Heating to 200°C leads to debrominated and polymerized ribbons that coexist with the oxide patches (Figure S5A). Further annealing above 300°C leads to apparent polymer cyclodehydrogenation, but also to more surface chemistry, such as the shrinking of the oxide patches (Figure S5B). We also observe that polymers located closer to the edge of an oxide patch are clearly distinct with significantly lower apparent height compared to the ones further away. We speculate that the atomic hydrogen from the dehydrogenation process effectively reduces the oxide layer. Moreover, GNRs grown in the vicinity of the oxide patches appear as depressions, presumably due to further dehydrogenation of their corner atoms.

In the second experiment, we tried to oxidize the surface after fully forming GNRs (same procedure as described in the experimental section of the main text). The resulting molecular structures (Figure S5C) appear similar to the lower apparent height structures in the above-described experiment (Figure S5B). This further points to oxidation and dehydrogenation of the GNRs as was reported previously [47,48].

We, however, believe that tailored tuning of preparation parameters for a specific molecular system can substantially improve the sample condition for desired functionality. We presume that the platform could be appealing for investigation of nanographene systems whose formation relies only on Ullmann coupling step without the cyclodehydrogenation or where the amount of released hydrogen is lower than for DBBA. Our choice for DBBA in the present work had been motivated

by its commercial availability, enabling a larger community to utilize this model system for their research.

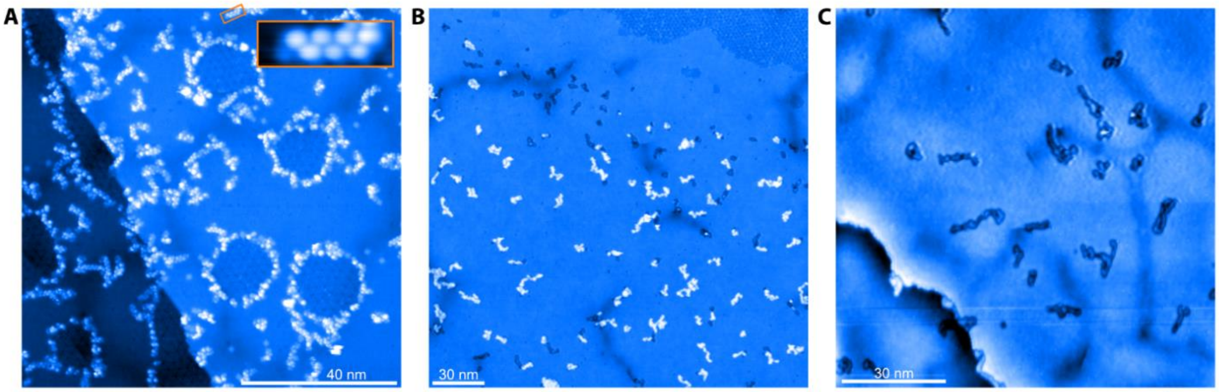


**Fig. S5** Overview topography images showing three different experiments aimed at investigating the coexistence of oxide patches and growth of 7-AGNR on $Cu_3Au$. **(A)** Deposition of DBBA on partially oxidized $Cu_3Au$ with subsequent annealing to 200 °C. **(B)** Deposition of DBBA on partially oxidized $Cu_3Au$ with subsequent annealing above 300 °C. **(C)** Oxidation of fully formed GNRs on $Cu_3Au$.

47. A. Berdonces-Layunta, J. Lawrence, S. Edalatmanesh, J. Castro-Esteban, T. Wang, M. S. G. Mohammed, L. Colazzo, D. Peña, P. Jelínek and D. G. de Oteyza, *ACS Nano*, 2021, **15**, 5610–5617.

48. J. Lawrence, A. Berdonces-Layunta, S. Edalatmanesh, J. Castro-Esteban, T. Wang, A. Jimenez-Martin, B. de la Torre, R. Castrillo-Bodero, P. Angulo-Portugal, M. S. G. Mohammed, A. Matěj, M. Vilas-Varela, F. Schiller, M. Corso, P. Jelinek, D. Peña and D. G. de Oteyza, *Nat. Chem.*, 2022, 1–8.
